# An Experimental Cold Gas Cannon for the Study of Porcine Lung Contusion and Behind Armor Blunt Trauma

**DOI:** 10.1007/s10439-023-03334-7

**Published:** 2023-08-02

**Authors:** Tomas Karlsson, Lars-Gunnar Olsson, Jenny Gustavsson, Ulf P. Arborelius, Mårten Risling, Mattias Günther

**Affiliations:** 1 Section of Experimental Traumatology, Department of Neuroscience, Karolinska Institutet, Biomedicum – 8B, 171 77 Stockholm, Sweden; 2 Section of Anesthesiology and Intensive Care, Department of Clinical Science and Education, Karolinska Institutet, Stockholm, Sweden

**Keywords:** Behind armor blunt trauma, Pulmonary contusion, Venous admixture, Porcine study, Gas cannon

## Abstract

Behind armor blunt trauma (BABT) is a non-penetrating injury caused by the rapid deformation of body armor, by a projectile, which may in extreme circumstances cause death. The understanding of the mechanisms is still low, in relation to what is needed for safety threshold levels. Few models of graded kinetic energy transfer to the body exist. We established an experimental model for graded BABT. The cold gas cannon was air-driven, consisted of a pressure vessel, a barrel, and a pressure actuator. It required short training to operate and was constructed by standard components. It produced standardized expulsion of plastic projectiles with 65 mm and weight 58 g. Velocity correlated linearly to pressure (*R* 0.9602, *p* < 0.0001), equation *Y* = 6.558*X + 46.50. Maximum tested pressure was 10 bar, velocity 110 m/s and kinetic energy (*E*_k_) 351 J. Crossbred male swine (*n* = 10) mean weight (SD) 56 ± 3 kg, were subjected to BABT, mean *E*_k_ (SD) 318 (61) J, to a fix point on the right lateral thorax. Pulmonary contusion was confirmed by physiological parameters pO_2_ (*p* < 0.05), SaO_2_ (*p* < 0.01), pCO_2_ (*p* < 0.01), etCO_2_ (*p* < 0.01), MPAP (*p* < 0.01), Cstat (*p* < 0.01), intrapulmonary shunt (*Q*′*s*/*Q*′*t*) (*p* < 0.05), and qualified trans-thoracic ultrasound (*p* < 0.0001). The consistent injury profile enabled for the addition of future experimental interventions.

**Supplementary Information:**

The online version contains supplementary material available at 10.1007/s10439-023-03334-7.

## Introduction

Behind armor blunt trauma (BABT) is a non-penetrating injury caused by the rapid deformation of body armor by a projectile hit [5]. When the body armor inhibits the projectile from penetrating the thorax, shock waves propagate and kinetic energy (*E*_k_) from the ballistic impact is transferred to the chest wall and to the internal organs, causing injury through rapid acceleration and deceleration of the tissue [18]. BABT may lead to cardiopulmonary failure and in extreme circumstances death [5, 6, 9]. Personal armors are specified to stop penetration from given types of ammunition. Since BABT injuries do exist, criteria for allowable back-face movement should also be used. Especially for high energy rifle ammunition general standards are still lacking. There is also a concern that BABT could be a more important problem when newer protection materials with a lower mass (areal density) provide the same efficiency against penetration. Increased research efforts of injury mechanisms are therefore needed to meet the increased risk of severe pulmonary injury [5, 6, 30]. BABT is well described in gelatin models but the correlation with human physiology is low [6, 19, 23, 29].

BABT injury mechanisms are complex and differ from other types of thoracic trauma, e.g., traffic accidents, and may include characteristics of primary blast injury [5]. It is not fully known how the lung injury correlates to the *E*_k_ of the projectile and the energy absorbed by the thorax. *E*_k_ is a form of energy that an object or a particle has by reason of its velocity and mass. Trauma by large objects impacting the thorax may result in elastic deformation which diverts energy to the thoracic cavity. Stopping a high energy bullet requires a hard-armor plate. When the bullet hits the plate both the bullet and plate material is disintegrated, and part of the incoming energy is transformed to heat. The total effect of the armor panel is therefore a reduction of *E*_k_, a high reduction of velocity and a spread of force over a larger area.

To control and vary input force characteristics we previously constructed a BABT-simulator [1] which was validated to a high velocity projectile assault rifle 7.62 mm bullet impacting an armor pack including a ceramic plate placed on the thorax at 800 m s^−1^, as previously described [10, 22, 23]. It consisted of a gun powder driven weapon with a Mauser-type cartridge case which shot 65 mm plastic projectiles, and the amount of gun powder was calibrated for desired velocity. While the device was effective, gun powder weapons may require permits and confined areas. Therefore, the aim of this study was to develop an air-driven launch device, which would be relatively simple, could be used after a short training and would be constructed using standard components. We hypothesized that the model may be useful for studies of BABT, in police and military settings.

## Material and Methods

### Air Cannon Construction Overview

**Fig. 1:**
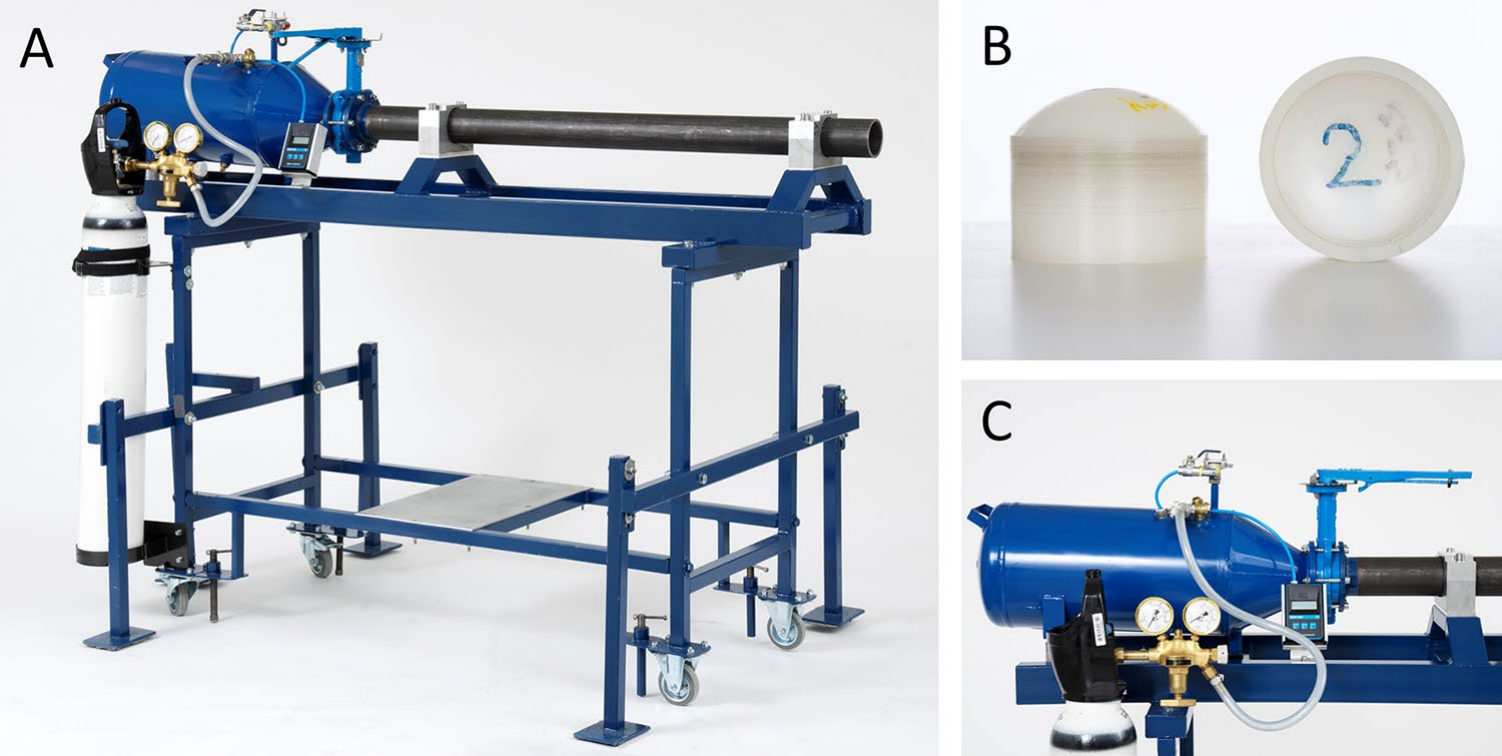
**A** Photo of the air cannon. **B** Photo of the 65 mm plastic projectiles. **C** Photo of the firing mechanism, including a pressure vessel, a barrel, and a pressure actuator. Note the handle (right) for manual turning of the butterfly valve for firing

The cannon consisted of a pressure vessel, a barrel and a pressure actuator placed between the pressure vessel and the barrel (Fig. 1). The pressure vessel was standard type with a volume of 24 L and made for 8 bar and tested at the manufacturer to 15 bar inflation pressure. The pressure vessel was fed with either a compressor or a pressurized tank. The barrel was a seamless cold-drawn precision steel pipe according to DIN 2391 with inner diameter of 65 (difference 0.01 to − 0.04) mm and wall thickness of 5 mm. The first tests were done with a 25 mm manually maneuverable ball valve which was connected through threaded joints and the later used torsion valve with through 8.8 bolts M8 × 80 mm. The shaft of the butterfly valve was milled out to obtain improved throughput. Later, a 65 mm manually operated torsion valve (maximum pressure 16 bar) was introduced (DN65 ALTECH VRID SPJ PN16HS). The valves were strapped in between welded flanges on the tank and barrel. The barrel was strapped with 50 mm thick stripes of high-strength aluminum (7075-T6). The stripes were internally clad with 1 mm rubber cloth. The stripes were bolted to a jumper with a profile of 60 × 30 × 2 mm (DIN 2395). The jumper was then welded to two parallel hole profiles (KKR SS 2134) 70 × 40 × 3 mm. The hole profiles had a length of about 1700 mm and outer measurement of 330 mm. The compressed air tank was adjusted in height and lined up with the torsion valve and barrel. Thick rubber plates were put between the brackets and tank to eliminate vibrations.

Three parameters were given from the previous studies: caliber 65 mm, projectile weight 58 g, with the same geometry and material as before, and projectile velocity at around 100 m s^−1^ [1]. The final version was preceded by three developmental versions. To understand considerations made, properties and designs of the other versions, we here describe all four versions. In versions 1 and 2, a compressor was used. In versions 3 and 4, it was replaced with gas supply from a compressed air tube. In version 4, one gable was cut off from the pressure tank and a conical part (length 140 mm) was welded to the tank. The lower part of the cone had an inner diameter of 68 mm which then in the connecting flange towards the butterfly valve coned down to 65 mm diameter. After welding the conical section, the tank was test-pressed to 10 bar. Beams and profiles included in the enclosure were hole profiles KKR 30 × 30 × 3 mm (SS2134). During development, it emerged that lateral stability needed to be improved which is why outriggers were added, which also provided better support against the floor. Two pairs of outriggers were placed sideways, which increased the width to about 1000 mm. A support leg was placed behind the tank to reduce recoil. All five outriggers were adjustable in height and equipped with a 100 × 70 mm support plate sheathed with a 5 mm thick rubber plate, to provide higher friction against the floor. Two swivel wheels at the rear and two fixed at the front allowed for transport of the cannon short distances. For further contact with the floor, 4 M16 × 1.5 mm threaded bars could be screwed to the floor. The center height of the barrel was 1085 mm. This was prompted by the height of the bunk where the swine was located. On top of the tank there were two ball valves; a 1/4ʺ valve for evacuation of air during projectile insertion and a 3/8ʺ valve connected by hose to the compressed air tube. An output was fitted for connection to a digital pressure gauge (Greisinger GDH 200-14) for a maximum of 11 bar. A muzzle adapter for the diameter of the barrel was made. The center hole was adapted for the use of a laser reticle (Site-Lite Laser Boresighter SL-150 Ultra Mag), normally inserted into the barrel of a rifle.

### Air Cannon Firing

Before each shot, a very fine-grained lamellar roundabout was driven rotating through the entire length of the barrel. After that, the roundabout was fitted with a drying paper moistened with alcohol and manually passed with a twisted movement back and forth in the barrel. This cleaning process removed rust flakes from the tank which could remain in the barrel from the previous shot. The torsion valve and the air evacuation valve were open when the projectile was inserted. The projectile was inserted until it stopped against the butterfly valve. After that, the two valves were closed. The ball valve for filling air was closed after the charging process. On completion of charging, the tank was filled with pressurized air. Firing was performed by manually rotating the firing valve handle (ball valve and later butterfly valve). Due to the large amount of air outflowing when the cannon was fired, an aperture was needed to avoid damage or displacement of equipment next to the animal. It consisted of a 90 × 210 × 5 cm board with a circular 100 × 110 mm hole. With the help of the laser sight, the board was adjusted so that the laser dot was within ± 1 mm of the center. With a mirror to the board, the laser beam was ensured to be reflected towards the mouth of the reticle, so that the thickness of the board did not adversely affect the projectile. It was of paramount importance that the laser beam was in the middle of the hole. The board was located 1.2 m from the opening of the barrel. The velocity of the projectile was measured at 2.2 m from the opening of the barrel, about 0.8 m from the swine. A speedometer with two light curtains with 300 mm distance in between was used. The projectile broke the light curtains, which gave the velocity measurement. In addition to keeping most of the air away from the swine, the aperture did also limit the airflow towards the speedometer, which could trigger the speedometer. We detected that an aperture was not needed at charging pressures below 5 bar, and that pressures between 8 and 10 bar could inflict errors in the velocity measurement, when the speedometer was triggered by the condensed air (shock wave). Advanced velocity calculations with respect to velocity and pressure were initially made from the version with 25 mm ball valve and showed that a 25 mm opening was too small and that the opening time needed to be significantly shortened. We found during the tests that low friction between the barrel and projectile was crucial for velocity. Overly "sluggish" projectiles resulted in lower velocity. Longer barrels generally provide opportunities for higher velocities in gunpowder-driven cannons. We did not try this in our cannon because of considerable modifications of the barrel support.

### Animal Preparation

The study was approved by, and conducted in accordance with, the Swedish ethics approval board for animal research (Approval No 12578-2020). The animals were housed in an accredited animal facility for up to 5 days prior to the experiment and fed a standard diet with free access to tap water. The ambient room temperature was maintained at 21–22 °C with 12-h light/12-h darkness cycles. The preparation was analogous to our earlier studies, including [1, 7, 14, 15, 22, 25]. In short, crossbred male specific pathogen free swine (Company Johansson, Stockholm Region, Sweden) with a mean weight (SD) 56 ± 3 kg, were premedicated with 150 mg tiletamine/zolazepam (Zoletil 100 Vet) and 6 mg medetomidine (Dormitor). After preoxygenation and assisted ventilation, anesthesia was induced in the right or left auricular vein, in a supine position on a standard operating table, with alfentanil 40 µg kg^−1^ and pentobarbitalnatrium 6 mg kg^−1^. Tracheal intubation was performed with a custom-made Miller-type laryngoscope and a Frova intubating catheter (Cook Medicals, Bloomington, IN, USA), using a standard cuffed size 8 tube (Rüsch, Teleflex, Morrisville, NC, USA). Anesthesia was maintained with ketamine 20 mg kg^−1^ h^−1^, midazolam 0.0485 mg kg^−1^ h^−1^. A 500 mL bolus of Ringer´s Acetate was given for 30 min. The animals were ventilated with a Hamilton C2 (Hamilton Medical, Geneva, Switzerland) using pressure synchronized intermittent mandatory ventilation with initial settings PEEP 4, PIP 15 cm H_2_0, respiratory rate 12 min^−1^, trigger flow 5 L min^−1^ and FiO_2_ 21%. Settings were continuously adjusted to achieve normoventilation and the ventilation was mechanical. Ventilation data was obtained from the ventilator. A 7.5 F Swan-Ganz pulmonary artery catheter (Edwards Lifescience) was introduced in the surgically exposed right external jugular vein and used for core temperature, central venous pressure (CVP), cardiac output and mixed venous oxygen saturation (SvO_2_). The right radial and femoral arteries were cannulated, ultra-sound assisted with a 20 G (Braun Medicals, Melsungen, Germany) and a 7 F catheter, respectively (Merit Medical, South Jordan, Utah, USA) and allowed for continuous blood pressure measurements, blood samples and blood removal. Arterial blood gases were taken continuously. Twelve ECG electrodes and a supra-pubic catheter were positioned. At completion of the experiments, the animals were euthanized with 70 mL pentobarbital (100 mg mL^−1^).

### Lung Contusion

The animals were subjected to chest trauma by pulmonary contusion by one shot of a 58 gram, 65 mm diameter × 55 mm length polyethylene projectile, deployed 3.3 m from the air cannon, producing an energy-burst to the thorax. Projectile velocity was mean (SD) 105 (10) m/s and *E*_k_ was mean (SD) 318 (61) J. The projectile hit a fix point on the right lateral thorax (5 cm caudal and 2 cm ventral to the tip of the right scapula, 22–24 cm dorsally of the xiphoid process, right front leg in maximal abducted position). We have earlier described lung contusion by similar trauma. In our experience, an increase in pulmonary shunt, in combination with positive findings in a qualified ultrasound of the lung (by a specialist physician in anesthesiology and intensive care) provide the most accurate evidence of lung injury, which we here utilized to describe the effects of the cannon [1, 7, 22].

### Ultrasound Protocol

A Piloter Ultrasound scanner with a Linear piezoelectric crystal alignment L10-5 (5-10 MHz) probe was used (Core Imaging, Grand Rapids, MI, USA). First, a pre-scan was performed over the intended impact zone before trauma, for baseline images. Second, a re-scan was performed over the injury zone after trauma to diagnose objective findings associated with lung contusion. The checklist included: 1. Lung slide: Yes/No, if No: Seashore or Barcode sign and lung point Yes/No. 2. Multiple B-lines/Confluent B-lines: Yes/No. 3. Z-lines, false B-lines, defined by (a) less echoic than the pleural line; (b) ill-defined; (c) short, vanishing after 2 to 4 cm, (d) not erasing the A-lines (horizontal arrows) (e) not moving with lung sliding. (Indicating healthy subject or pneumothorax) [17]: Yes/No. 4. Thick pleural line: Yes/No. 5. Irregular pleural line: Yes/No. 6. Hypoechoic localized pleural effusion: Yes/No. 7. Broken ribs (probe in transverse and horizontal): Yes/No [4, 8].

### Calculations

The *E*_k_ of the projectiles was calculated from the *E*_k_ equation, where *V*_0_ was the terminal velocity of the projectile before impact and m was the mass of the projectile.

$$E_{{\text{k}}} = V_{0}^{2} \times m/2$$

Venous admixture (*Q*′*s*/*Q*′*t*) is the calculated estimate of how much hypoxic blood would be required to produce the measured arterial oxygen (O_2_) results, for a given cardiac output. It is a measure of how much blood is “shunted” past the lung without being oxygenated. It was calculated by the shunt equation (Berggren equation) where *Q*′*s*/ *Q*′*t* = shunt fraction (shunt flow divided by total cardiac output), CcO_2_ = pulmonary end capillary O_2_ content, same as alveolar O_2_ content = SaO_2_ × Hb × 1.3. SaO_2_ is assumed at 100% in the lungs. CaO_2_ = arterial O_2_ content (SaO_2_ × Hb × 1.3), CvO_2_ = mixed venous O_2_ content (SvO_2_ × Hb × 1.3). *V*′*A*/*Q*′ = alveolar minute ventilation/cardiac output. Units: CcO_2_/CaO_2_/CvO_2 =_ mL L^−1^, Hb = g L^−1^, SaO_2_ = % [27].

$$Q^{\prime}s/Q^{\prime}t \, = \, \left( {{\text{CcO}}_{2} - {\text{CaO}}_{2} } \right)/\left( {{\text{CcO}}_{2} - {\text{CvO}}_{2} } \right)$$

### Statistical Analyses

Statistical analyses were done using GraphPad Prism version 9.5.1 (GraphPad Software). Baseline values were collected at 0 h. Cannon pressure and resulting projective velocity (m s^−1^) were fitted with linear regression with predictor pressure (bar). The linear regressions were performed to understand this relationship for each version of the device. Physiological consequences of lung contusion were analyzed using paired two-tailed t-tests. These were performed to understand changes in lung physiology before and after discharge of the projectile. Pulmonary ultrasound findings were analyzed using Chi-square. These were performed to understand lung injuries detected by ultrasound before and after discharge of the projectile. The primary outcome was *Q*′*s*/*Q*′*t*. Error bars display the standard deviation. *p* < 0.05 was considered significant.

## Results

**Fig. 2:**
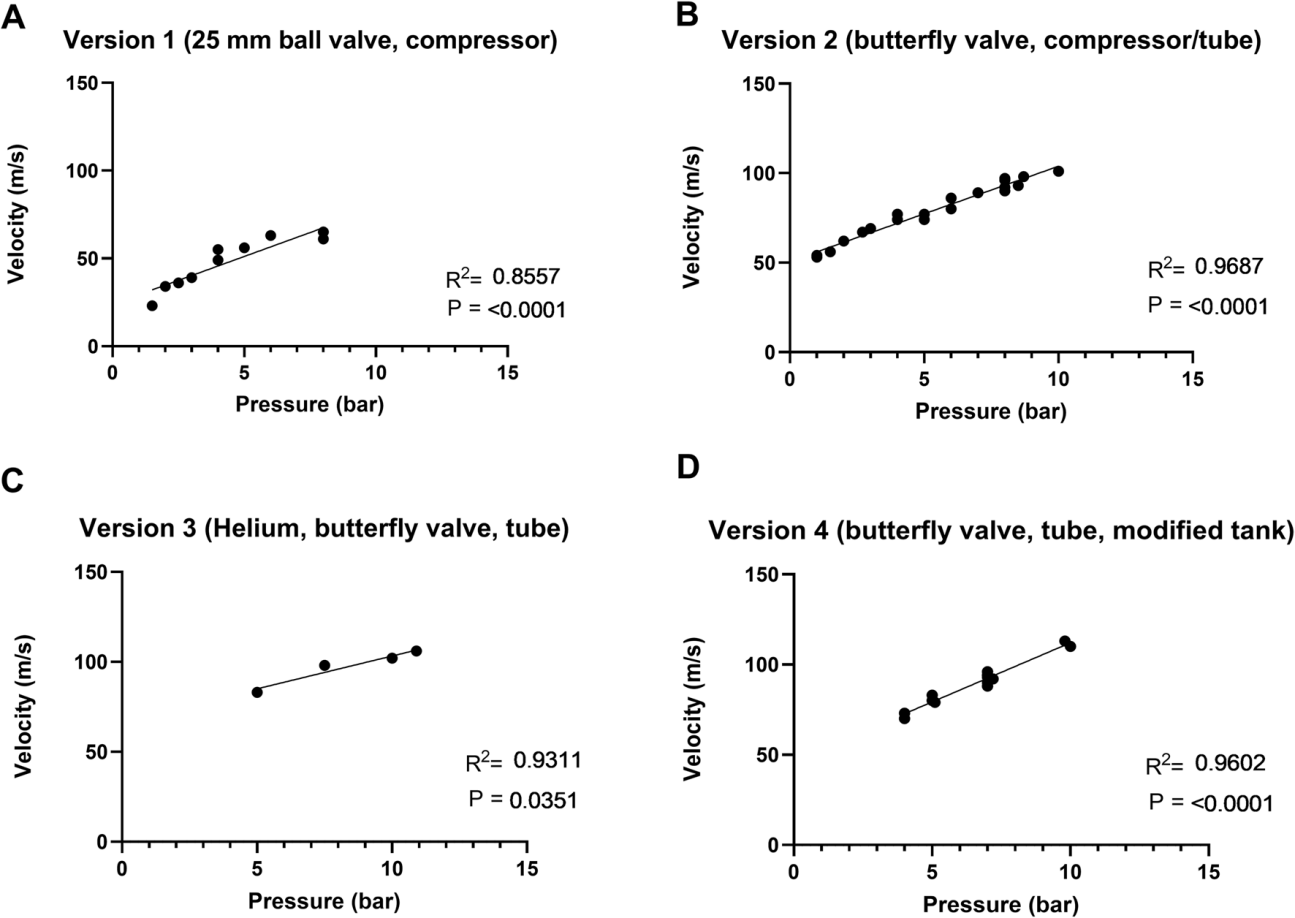
Linear regression analyses of velocity as a function of pressure, in four developmental versions of the air cannon. **A** version 1 using a ball valve and compressor. **B** version 2 using a butterfly valve and compressor/tube. **C** version 3 using a butterfly valve and gas supply from a helium tube. **D** version 4 as version 2 but with modified pressure tank (one gable was cut off from the pressure tank and a conical part with length 140 mm was welded to the tank). The equation for version 4 (final version) was *Y* = 6.558*X + 46.50.

The air cannon was made in four developmental versions and evaluated with linear regression analyses of projectile velocity as a function of driving pressure. Version 1 was made with a 25 mm ball valve, and air supply from a compressor. Velocity correlated linearly to pressure (*R* 0.8557, *p* < 0.0001). The equation was *Y* = 5.445*X + 23.90 (Fig. 2A). Pressures above 6 bar did not increase velocity because of a small valve aperture and the limit was estimated to about 65 m s^−1^. Version 2 was made with a butterfly valve and air supply from a compressor and tube, as the velocities did not differ between gas supply methods. Velocity correlated linearly to pressure (*R* 0.9687, *p* < 0.0001). The equation was *Y* = 5.316*X + 50.71 (Fig. 2B). Version 3 was made with a butterfly valve and gas supply from a helium tube. Velocity correlated linearly to pressure (*R* 0.9311, *p* < 0.05). The equation was *Y* = 3.649*X + 66.78 (Fig. 2C). Version 4 was made with a butterfly valve and air supply from a pressurized tube and a modified tank (one gable was cut off from the pressure tank and a conical part with length 140 mm was welded to the tank). Velocity correlated linearly to pressure (*R* 0.9602, *p* < 0.0001). The equation was *Y* = 6.558*X + 46.50 (Fig. 2D). Detailed data are provided in Supplemental Fig. 1.

**Fig. 3:**
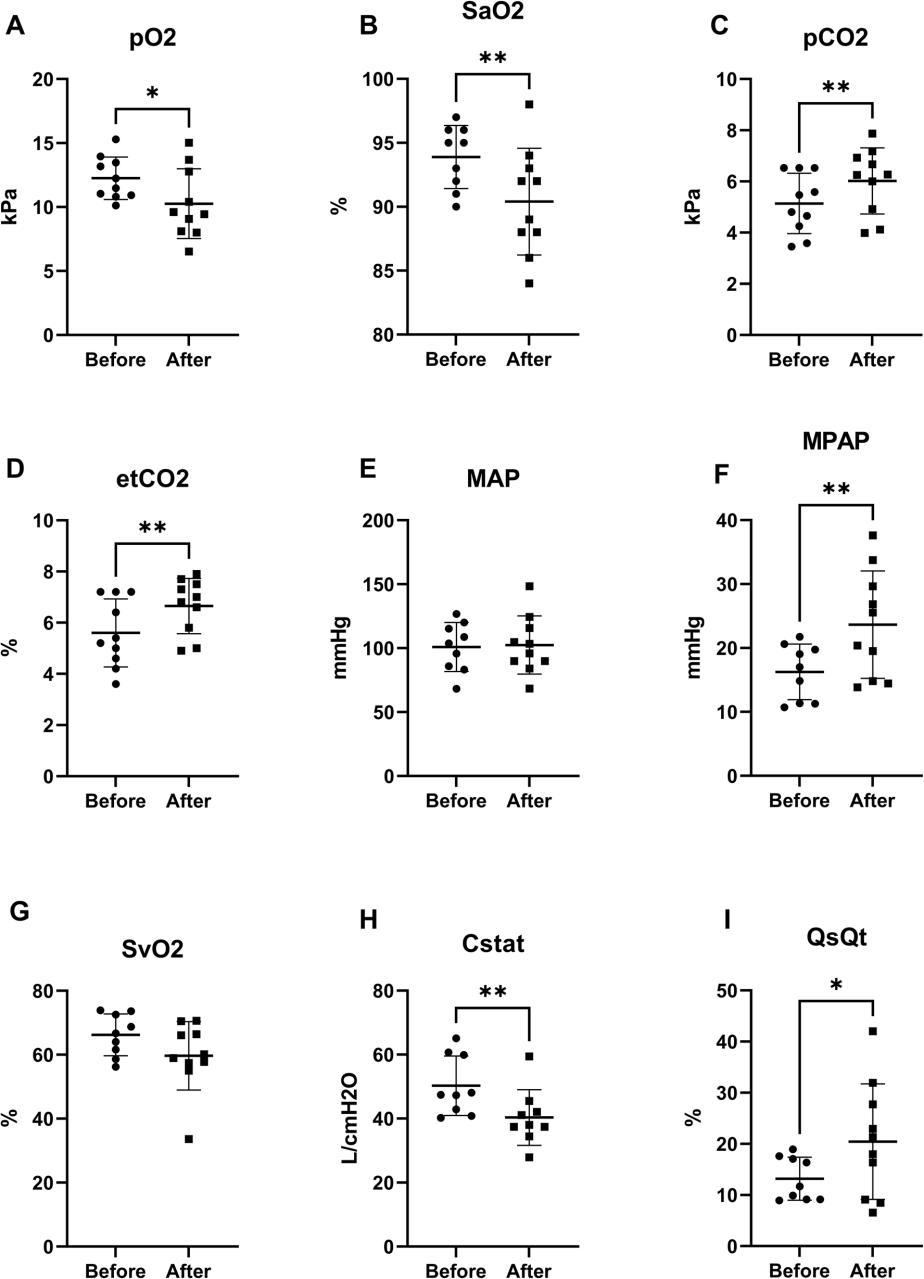
Physiological consequences of lung contusion by the air cannon. Values taken at baseline before the shot and at 15 min after the shot. **A** and **B** arterial pO_2_ and hemoglobin-oxygen saturation (SaO_2_) decreased. **C** and **D** arterial pCO_2_ and end-tidal CO_2_ increased. **E** mean arterial pressure (MAP) was unaffected. **F** mean pulmonary arterial pressure (MPAP) increased. **G** mixed venous saturation (SvO_2_) was unaffected. **H** static compliance (Cstat) of the lung decreased. **I** intrapulmonary shunt (*Q*′*s*/*Q*′*t*) increased. *p < 0.05, **p < 0.01

The following physiological findings were identified at 15 min after the event, in comparison to baseline. Increases were detected in arterial pCO_2_ (*p* < 0.01) (Fig. 3C), end-tidal CO_2_ (*p* < 0.01) (Fig. 3D), MPAP (*p* < 0.01) (Fig. 3F) and *Q*′*s*/*Q*′*t* (*p* < 0.05) (Fig. 3I). Decreases were detected in arterial pO_2_ (*p* < 0.05) (Fig. 3A), SaO2 (arterial hemoglobin-oxygen saturation) (*p* < 0.01) (Fig. 3B), and static compliance of the lung (Cstat) (*p* < 0.01) (Fig. 3H). No changes were detected in MAP (Fig. 3E) or SvO_2_ (Fig. 3G).

**Fig. 4:**
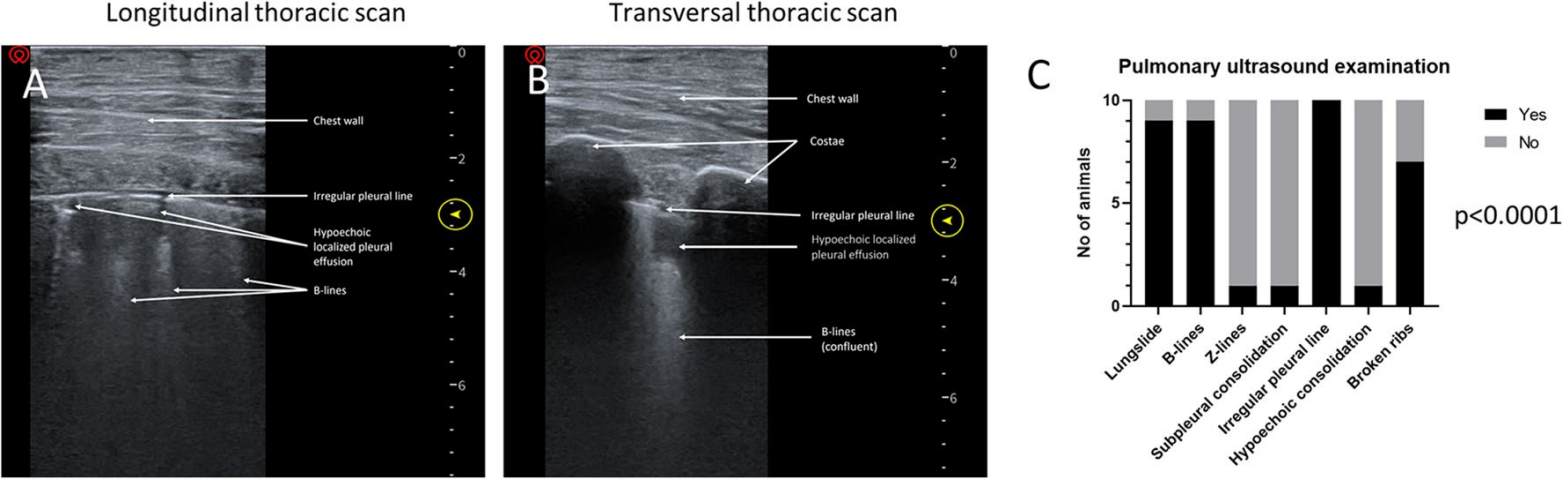
Pulmonary ultrasound examination after lung trauma by the air cannon, confirming changes in lung pathology. None of the changes were detected in the pre-scan. *p* < 0.0001

The following positive physiological findings were identified after the event via longitudinal and transversal thoracic ultrasound. The percentage of positive findings for injury were: Absent lungslide 10%, B-lines 90%, Z-lines 10%, subpleural consolidation 10%, irregular pleural line 100%, hypoechoic consolidation 10% and broken ribs 70% (p < 0.0001). None of the changes were detected in the pre-scan before contusion (Fig. 4A–C).

## Discussion

In this study we described an experimental model for thoracic impact, using an air-driven 65 mm canon which enabled controlled and varied input force characteristics of BABT. Using the model, we demonstrated lung contusion injuries by physiologic parameters and trans-thoracic ultrasound.

The most used safety standard for body armor BABT prevention in both police and military applications is based on a maximum back-face signature of 44-mm in clay (Roma Plastilina 1). It is based on a relationship between goat-thorax deformation, when covered with armor and exposed to pistol shots, and probability of lethality [11]. The standard was meant to be preliminary at the time of conception in the 1970 s, and not to have the widespread use it has today. The NIJ (National Institute of Justice, USA) BABT standard has also been used in applications for which it was never intended nor validated [11]. Examples of unintended use include the assessment of hard-body armor, body armor for small individuals, and impacts with rounds with much higher velocity and *E*_k_ than the pistol ammunition used in the original investigation. Impacted hard-armor plates load the torso differently than soft body armor, which could create different injury patterns in BABT situations [11]. Therefore, to enable improved predictions of safety, current research on BABT injuries should investigate the physiologic consequences of high velocity BABT and hard plate armors. Measuring the absorbed *E*_k_ is methodologically challenging, and investigations of porcine chest trauma by bolt guns have not assessed graded injury [2, 3].

We previously described a gun powder driven BABT-simulator, which enabled studies of graded BABT injury [1], which was simple in construction but complicated to operate accurately. Also, it was difficult to decrease the velocity of the projectile linearly by decreasing the amount of gun powder. To further lower the threshold for conducting studies on BABT, we constructed an air-driven model, which would not require gun permits or confined laboratories. The air cannon was made in four developmental versions. It emerged that there was not much difference in velocity if the air filling was done from the compressor or from the air tube (version 2). When air is compressed, the temperature of the air increases and produces the same pressure with a lower amount (mass) of gas, which tend to give a lower resulting velocity. Air from the tube expands when filling and becomes colder (adiabatic process). For this reason, air from the tube should theoretically provide higher velocities. However, the flow of air was not optimal which is why the difference between compressor air and tube air became negligible. We found that with repeated firing using compressor air (two shots in close succession), the velocity decreased with each repetition. Thus, it was an advantage to fill with cold air from the tube. In version 3, helium was tested as propellant. Air has a high molecular weight compared to helium, and accelerating air up to the desired velocity of the projectile requires energy. With helium, only minor velocity improvements were detected, likely because of the design of the cannon and possible imperfect gas flow conditions, which is why the higher costs to use helium were not justified. To improve the flow of air through the butterfly valve, a conical transition was made (version 4), which improved the velocity in relation to pressure. This version produced adequate projectile velocities and could decrease and increase velocities within the desired pressure loading range, and thus became the final version with which we investigated pulmonary consequences in vivo in swine. The final version was based on standard components as the intention was not to make the cannon overly complicated. For safety reasons, we limited the total pressure to 10 bar. Ideally, it may be possible to increase pressure and decrease the tank size. However, this adds risk. Several shots with repeated loads increase tension on threaded joints and material fatigue can occur and cause accidents. However, the effect of repeated shots on material fatigue was not investigated and should be the focus of further investigations.

In future developments, new devices for measuring velocity are under testing and thus reducing the problems with the influence from the shock wave. In different types of experiments, we have connected an oscilloscope to the speedometer to be able to distinguish projectile and shock wave velocities. An opening valve that is remotely controlled with electricity or pneumatics would be desirable. The opening time would always be the same and not depend on operator. However, it is difficult to find a remote-controlled 65 mm valve that is fast enough. An alternative method for gas release is to use frangible membranes. This method has also been used in shock tubes. One membrane could hold a certain pressure, and a pressure near this limit could be produced, and then released to accelerate a projectile, when the membrane is punctured. More than one membrane in a series could be used to add more pressure for each membrane. This technique may result in shorter opening time but requires extensive design and testing of suitable membranes.

The intention of the cold gas cannon was to create lung contusions similar to those previously described [1]. Therefore, we investigated physiological responses and trans-thoracic ultrasound. Pulmonary contusion is characterized by the presence of the following: (1) alveolo-interstitial syndrome (AIS). AIS is defined in ultrasound as the existence of multiple, or an increase in B-lines arising from the pleural line in a patient with no clinical suspicion of cardiogenic pulmonary edema, or by (2) a peripheral parenchymal lesion (PPL), defined as parenchymal disruption with localized pleural effusion with or without disintegrated pleural line or consolidations (“hepatization”) [16]. The ultrasound examinations were performed by a specialist in anesthesiology and intensive care, using a 5-10 MHz transducer. Lung ultrasound is normally best performed with a low-frequency curvilinear transducer (3-6 MHz), but there is also an advantage in using a high-frequency linear transducer (7-12 MHz) to visualize lung slides, the pleura in higher resolution, superficial structures, and A-lines [4, 8]. Our experience is that these features are important in the immediate diagnostics after the lung trauma model. After an initial blunt or blast thoracic trauma, an edematous phase deteriorates the interstitial edema within the first 1–2 h after injury. The air spaces become inundated with blood, inflammatory markers, and tissue debris, as there is an increase in alveolar and capillary permeability along with a reduction in surfactant production. Within 24–48 h after the onset of injury, there is alveolar collapse and further consolidation due to the extravasation of blood into the alveoli. Lung consolidation can lead to increased vascular pressures causing pulmonary hypertension and retention of blood. The resulting ventilation/perfusion mismatch, increased pulmonary shunting, decreased gas exchange, and decreased compliance can predispose patients to clinically apparent symptoms such as hypoxia, hypercarbia (increased carbon dioxide levels in the blood), tachypnea, hemoptysis and wheezing. These mechanisms of consolidation, shunting and mismatch also predispose patients with pulmonary contusions to pneumonia and acute respiratory distress syndrome (ARDS) [21]. Ultrasound examination has a sensitivity of 94% and a specificity of 96% for lung contusion [12, 26].

We assessed physiological consequences at 15 min after impact. We have shown that most consequences, if survivable, peak within 15 min and are reversible within 1 h [22]. We specifically assessed markers of pulmonary gas exchange and circulation: pO_2_, SvO_2_, MAP, MPAP, SaO_2_, pCO_2_, etCO_2_, Cstat and venous admixture (*Q*′*s*/*Q*′*t*). In our experience, these physiological measurements are the most affected by BABT, and it is likely that lethality from high velocity BABT is a consequence of early and severe gas exchange impairments. The hypoxia is related to a severe and transiently increased venous admixture (*Q*′*s*/*Q*′*t*) in the exposed lung. We detected characteristic changes in pO_2_, SaO_2_, pCO_2_, etCO_2_, MPAP and *Q*′*s*/*Q*′*t*, which confirmed the pulmonary injury [10, 22]. In addition, we detected a decrease in Cstat. Cstat is the compliance of the lung, which is defined as the change in volume divided by the change in pressure [20].

Some limitations need to be discussed. First, the observation time was limited to 15 min. Respiratory mechanics are affected early after pulmonary contusion [21], which is why we deemed this time span appropriate for evaluation of acute changes in lung function. However, future studies may include longer observation times to investigate delayed effects of BABT trauma, which may be characterized by lung hemorrhage, alveolar collapse, pulmonary edema and an inflammatory response [21, 24, 28] but were beyond the scope of this investigation. Second, the similar size and anatomy of swine organs and human organs allow the model to be particularly beneficial for translational research. In the field of respiratory medicine, the similarities between swine and human lungs give porcine models the potential of advancing translational medicine. However, interspecies differences in lung function, such as decreased pulmonary compliance in swine, may limit extrapolation to humans with BABT [13, 31]. Third, only the final version of the cannon was tested in vivo, which decreased the number of animals required to be sacrificed. Therefore, pulmonary consequences of earlier developmental versions were not evaluated.

The pulmonary consequences by BABT display unique features compared to other forms of experimental pulmonary contusion. BABT-specific models are therefore needed for adequate assessments of injuries related to protective equipment. Body armors validated for handgun- and grenade fragment protection are still used, where high velocity weapons are a realistic threat, and few reports include data of energy levels equal to- or higher than 7.62 mm rifle bullets [6]. Light body armors may not provide enough protection from the *E*_k_ transferred from a high energy projectile and the development and increased use of light-weight body armors may increase injuries [9]. Body armor designs and injury assessments may therefore be initially guided by relevant BABT-models to encompass the severe injury spectrum from high velocity projectile BABT. This BABT-simulator allowed for improved assessments of the physiological response of BABT. We believe that the cannon may also be useful for studies of behind helmet blunt trauma (BHBT) in swine models and that the device may facilitate the development of body armor and helmets, to meet the requirements of future ballistic safety equipment and the development of new BABT and BHBT safety criteria. The blunt trauma dose may be increased by higher impactor mass or increased striking velocities. It is also possible to modify the system to simulate BABT sustained while wearing soft armor instead of hard armor, using different impactor nose shapes (e.g., conical), which would need to be validated in future studies.

### Supplementary Information

Below is the link to the electronic supplementary material.

Supplementary file1 (PDF 151 kb)

## Data Availability

The data that support the findings of this study are available from the corresponding author upon reasonable request.
