# Supplementary material for: An Experimental Cold Gas Cannon for the Study of Porcine Lung Contusion and Behind Armor Blunt Trauma
Source: Ann Biomed Eng. 2023 Aug 2;51(12):2762–71. doi: 10.1007/s10439-023-03334-7 (PMC10632235; doi:10.1007/s10439-023-03334-7)
Supplement: Supplementary file 1 — Supplementary file1 (PDF 151 kb) [file 10439_2023_3334_MOESM1_ESM.pdf]

Supplemental Figure 1

SUPPLEMEMENTAL FIGURE 1

Version 1

Loading pressure (bar)	Velocity (m/s)	Mass (g)	Ek (J)
1,5	23	58	15,341
2	34	58	33,524
2,5	36	58	37,584
3	39	58	44,109
4	55	58	87,725
4	49	58	69,629
5	56	58	90,944
6	63	58	115,101
8	61	58	107,909
8	65	58	122,525
8	65	58	122,525

	Velocity	Ek
Minimum	23	15,34
25% Percentile	36	37,58
Median	55	87,73
75% Percentile	63	115,1
Maximum	65	122,5
Range	42	107,2
10% Percentile	25,2	18,98
90% Percentile	65	122,5
95% CI of median		
Actual confidence level	98,83%	98,83%
Lower confidence limit	34	33,52
Upper confidence limit	65	122,5
Mean	49,64	76,99
Std. Deviation	14,5	39,04
Std. Error of Mean	4,372	11,77
Lower 95% CI of mean	39,9	50,76
Upper 95% CI of mean	59,38	103,2

Version 2

Loading pressure (bar)	Velocity (m/s)	Mass (g)	Ek (J)
1	53	58	81,461
1	54	58	84,564
1,5	56	58	90,944
2	62	58	111,476
2,7	67	58	130,181
3	69	58	138,069
4	74	58	158,804
4	77	58	171,941
5	74	58	158,804
5	77	58	171,941
6	80	58	185,6
6	86	58	214,484
7	89	58	229,709
8	90	58	234,9
8	92	58	245,456
8	96	58	267,264
8	97	58	272,861
8,5	93	58	250,821
8,7	98	58	278,516
10	101	58	295,829

	Velocity	Ek
Minimum	53	81,46
25% Percentile	67,5	132,2
Median	78,5	178,8
75% Percentile	92,75	249,5
Maximum	101	295,8
Range	48	214,4
10% Percentile	54,2	85,2
90% Percentile	97,9	278
95% CI of median		
Actual confidence level	95,86%	95,86%
Lower confidence limit	69	138,1
Upper confidence limit	92	245,5
Mean	79,25	188,7
Std. Deviation	15,41	69,04
Std. Error of Mean	3,446	15,44
Lower 95% CI of mean	72,04	156,4
Upper 95% CI of mean	86,46	221

Version 3

Loading pressure (bar)	Velocity (m/s)	Mass (g)	Ek (J)
5	83	58	199,781
7,5	98	58	278,516
10	102	58	301,716
10,9	106	58	325,844

	Velocity	Ek
Minimum	83	199,8
25% Percentile	86,75	219,5
Median	100	290,1
75% Percentile	105	319,8
Maximum	106	325,8
Range	23	126,1
10% Percentile	83	199,8
90% Percentile	106	325,8
95% CI of median		
Actual confidence level	87,50%	87,50%
Lower confidence limit	83	199,8
Upper confidence limit	106	325,8
Mean	97,25	276,5
Std. Deviation	10,05	54,65
Std. Error of Mean	5,023	27,33
Lower 95% CI of mean	81,27	189,5
Upper 95% CI of mean	113,2	363,4

Version 3

Loading pressure (bar)	Velocity (m/s)	Mass (g)	Ek (J)
4	70	58	142,1
4	73	58	154,541
5,1	79	58	180,989
5	80	58	185,6
5	83	58	199,781
7	88	58	224,576
7	90	58	234,9
7	93	58	250,821
7	94	58	256,244
7	94	58	256,244
7	96	58	267,264
7,2	92	58	245,456
9,8	113	58	370,301
10	110	58	350,9

	Velocity	Ek
Minimum	70	142,1
25% Percentile	79,75	184,4
Median	91	240,2
75% Percentile	94,5	259
Maximum	113	370,3
Range	43	228,2
10% Percentile	71,5	148,3
90% Percentile	111,5	360,6
95% CI of median		
Actual confidence level	98,71%	98,71%
Lower confidence limit	79	181
Upper confidence limit	96	267,3
Mean	89,64	237,1
Std. Deviation	12,31	65,51
Std. Error of Mean	3,291	17,51
Lower 95% CI of mean	82,53	199,3
Upper 95% CI of mean	96,75	274,9
